# ﻿Taxonomic review of the genus *Zanchius* Distant, 1904 (Hemiptera, Heteroptera, Miridae) from Vietnam, with the description of a new species and notes on host sharing

**DOI:** 10.3897/zookeys.1238.144351

**Published:** 2025-05-08

**Authors:** Junggon Kim, Tosaphol Saetung Keetapithchayakul, Quoc Toan Phan, Sunghoon Jung

**Affiliations:** 1 The Center for Entomology & Parasitology Research, College of Medicine and Pharmacy, Duy Tan University, Da Nang 550000, Vietnam; 2 Laboratory of Systematic Entomology, Department of Applied Biology, College of Agriculture and Life Sciences, Chungnam National University, Daejeon, Republic of Korea; 3 Department of Smart Agriculture Systems, College of Agriculture and Life Sciences, Chungnam National University, Daejeon, Republic of Korea

**Keywords:** Oriental Region, Orthotylinae, plant bugs, Southeast Asia, taxonomy, true bugs

## Abstract

The orthotyline plant bug genus *Zanchius* Distant, 1904 is taxonomically reviewed from Vietnam for the first time with the description of a new species, *Zanchiustuehang* Kim & Jung, **sp. nov.** Detailed descriptions and diagnoses of the species are provided, along with a key to the Vietnamese *Zanchius* species, supported by illustrations and photographs. Notes on host sharing, associations with auchenorrhynchan species, and potential distributions are also included.

## ﻿Introduction

The genus *Zanchius* Distant, 1904 is among the larger groups within the subfamily Orthotylinae (Hemiptera, Miridae), consisting of more than 40 described species (Schuh 2002–2013; Aukema et al. 2013). Morphologically, this genus is principally characterized by the following combination of characters: a generally spindle-shaped, shiny, greenish dorsum, which is sparsely or normally covered with shiny setae; a short, vertical head; a projected frons; protuberant compound eyes positioned forward on the head and distinctly separate from the anterior margin of the pronotum; and a wide, rounded hemelytron (Schuh 1974; Yasunaga 1999). The majority of *Zanchius* species have been recorded from the Afrotropic, Oriental, and Palaearctic regions (Linnavuori 1973, 1994; Schuh 1974, 2002–2013; Liu and Zheng 1999; Yasunaga 1999; Kim and Jung 2017; Yasunaga and Duwal 2017).

Biological information on this genus remains limited. However, it is well established that certain *Zanchius* species are associated with host plants and are assumed to act as predators of auchenorrynchan species found on the plants (Zheng and Liu 1993; Yasunaga 1999; Wheeler 2001; Kim and Jung 2017).

In Vietnam, no comprehensive taxonomic study or detailed information on the genus *Zanchius* exist, apart from a single examined *Zanchius* species based on a female specimen collected in Vietnam, as documented in Liu and Zheng’s (2014) book on Orthotylinae in China. This minimal and fragmented information has resulted in the underrepresentation of the genus in regional records.

This study presents the first taxonomic review of the orthotyline genus *Zanchius* in Vietnam. Four species are recognized, including a new species and one additional record. Detailed morphological information, including descriptions and diagnoses of each species, as well as a key to the *Zanchius* species in Vietnam, is provided. Habitat sharing, associations with auchenorrynchan species is briefly discussed.

## ﻿Materials and methods

Photographs of the examined specimens were taken using a ZEISS Stemi 508 stereomicroscope equipped with an OPTIKA C-P6 digital camera. The figures were prepared using Adobe Photoshop 2020. Illustrations were created using the Procreate application on an iPad Pro 2020, based on representative digital photographs. Final plates were assembled using Affinity Photo v. 3. All measurements provided are in millimeters (mm) and were taken using ToupView software installed on the same camera (Table 1). To examine male and female genitalia, the abdomen was detached and immersed in a 10% KOH solution for 5 min at 70 °C until the internal structures became visible. The type specimens are deposited in the Zoological Collection of Duy Tan University (**ZCDTU**), Da Nang, Vietnam. Terminology for external and genital morphological structures primarily follows Yasunaga and Duwal (2017). Distribution record and a host plant marked with an asterisk (*) indicate new records for the species in the area.

**Table 1.** Measurements of *Zanchius* species used in this study. Abbreviations: AS – antennal segment.

	*Z.tuehang* sp. nov.				*Z.marmoratus*						*Z.quinquemaculatus*	*Zanchius* sp.		
	Male *n* = 1	Female *n* = 3			Male *n* = 7			Female *n* = 10			Male *n* = 1	Female *n* = 4		
		Min	Max	Mean	Min	Max	Mean	Min	Max	Mean		Min	Max	Mean
Body length (clypeus-apex of membrane)	2.91	3.01	3.03	3.02	3.29	3.32	3.30	3.30	3.36	3.33	3.12	2.71	2.74	2.73
Head length (excluding collar)	0.25	0.26	0.27	0.26	0.30	0.31	0.31	0.31	0.33	0.32	0.26	0.24	0.24	0.24
Head width (including compound eye)	0.52	0.54	0.56	0.55	0.58	0.60	0.59	0.69	0.72	0.71	0.56	0.48	0.49	0.49
Vertex width	0.18	0.24	0.25	0.24	0.24	0.25	0.24	0.23	0.25	0.24	0.21	0.21	0.22	0.22
AS I	0.33	0.37	0.38	0.37	0.35	0.36	0.35	0.39	0.40	0.40	0.21	0.20	0.20	0.20
AS II	0.99	1.01	1.06	1.03	1.36	1.39	1.38	1.57	1.60	1.58	1.12	1.17	1.19	1.18
AS III	0.53	0.56	0.57	0.56	0.79	0.82	0.81	0.91	0.96	0.94	1.11	0.58	0.59	0.59
AS IV	0.43	0.45	0.47	0.46	0.71	0.72	0.72	0.87	0.90	0.88	0.81	0.54	0.55	0.55
Total AS length	2.28	2.39	2.48	2.43	3.21	3.32	3.26	3.74	3.86	3.80	3.26	2.49	2.53	2.52
Pronotum mesial length	0.30	0.32	0.33	0.32	0.35	0.36	0.35	0.44	0.47	0.46	0.30	0.28	0.29	0.29
Posterior pronotal width	0.84	0.87	0.89	0.88	0.82	0.85	0.83	0.93	0.97	0.95	0.72	0.67	0.70	0.69
Anterior scutellar width	0.61	0.64	0.65	0.65	0.61	0.63	0.62	0.73	0.77	0.75	0.53	0.52	0.53	0.53
Scutellum mesial length	0.43	0.48	0.49	0.48	0.51	0.53	0.52	0.59	0.63	0.61	0.52	0.37	0.38	0.38
Commissure length	0.81	0.85	0.88	0.86	0.92	0.94	0.93	1.22	1.29	1.26	0.94	0.75	0.77	0.77
Hemelytron (maximal width)	0.55	0.58	0.59	0.58	0.58	0.60	0.59	0.67	0.69	0.68	0.47	0.43	0.44	0.44

## ﻿Taxonomy

### ﻿Genus Zanchius

Taxon classificationAnimaliaHemipteraMiridae

Distant, 1904

CDE4E6DB-DC9E-5D97-A5B2-F9B749F4F9A9

Zanchius Distant, 1904: 477. Type species: Zanchiusannulatus Distant, 1904.
Zonodorus Distant, 1909: 522 (syn. Carvalho 1952: 79).
Uzeliella Poppius, 1911: 31 (syn. Carvalho 1952: 79).
Poppiella Bergroth, 1911: 188 (new name for Uzeliella Poppius, 1911).
Habrocoris Wagner, 1951: 153 (syn. Linnavuori 1964: 329).

#### Diagnosis.

Differs from other orthotyline genera by dorsum being generally greenish, shiny, spindle-shaped, sparsely or normally covered with shiny setae (vs dorsum being gradually widened caudad; *Latizanchius* Liu & Zheng, 2001); head short and vertical; frons convex; clypeus not or only barely visible dorsally; compound eyes protuberant, set forward on head, removed from anterior pronotal margin; first antennal segment distinctly short, shorter than head width across eyes; exposed part of mesoscutum wide; hemelytra hyaline or subhyaline, wide and rounded; cuneus somewhat small relative to corium (updated from Schuh (1974) and Yasunaga (1999)).

#### Discussion.

The genus *Zanchius* has traditionally been diagnosed based on external morphology, particularly its greenish dorsum and head structure with compound eyes that are conspicuously set forward. Species exhibiting these characters have been assigned to *Zanchius* within Orthotylini. However, the diversity in pygophore structure and the male and female genitalia (e.g. Figs 2, 3) within *Zanchius* raises questions regarding its monophyly.

Additionally, Liu and Zheng (2001) established the genus *Latizanchius*, primarily distinguishing it from *Zanchius* based on differences in body shape and the ratio between the first antennal segment and head width. Notably, the genital structures of *Latizanchius* also exhibit significant variation (Liu and Zheng 2001).

*Zanchius* is a species-rich group within Orthotylini, and the female genitalia are scarcely studied. As the need for monophylogical assessment has already been emphasized for other genera of Orthotylinae (e.g. *Pseudoloxops* Kirkaldy, 1905; see Yasunaga et al. 2022; Kim et al. 2024), a similar evaluation is required for *Zanchius*. Future studies should test the monophyly of *Zanchius* and its allied genera, as well as reassess the placement of species currently assigned to *Zanchius*.

After the recognition of natural groups within Orthotylinae (e.g. the *Falconia*, *Zanchius* groups) by Schuh (1974), it has been proposed that the *Zanchius* group should be upgraded to a new tribal level, as it possesses distinct morphological characters that differentiate it from the traditional Orthotylini (e.g. a dorsally flattened head with small, anteriorly directed eyes set apart from the pronotum, delicate and often semitransparent forewings; Yasunaga and Duwal 2017).

Recently, Bolshakova and Konstantinov (2022) provided a molecular phylogeny of the Orthotylinae, revealing that the *Zanchius* group is indeed monophyletic and separated from other Orthotylini members. Additionally, Bolshakova and Konstantinov (2022) presented the morphological characters of its representatives.

In our study, we conducted a review of the genus *Zanchius* in Vietnam, providing not only previously known morphological features but also new insights into the largely unstudied female genitalia. Notably, the interramal lobe extends far beyond the interramal sclerite (Fig. 3B, D), a character shared with other members of the *Zanchius* group (e.g. *Malacocoris* Fieber, 1858 and *Filicicapsus* Bolshakova & Konstantinov, 2022; see Bolshakova and Konstantinov 2022: fig. 6b, d). Although further examination of female genitalia from a greater number of species within *Zanchius* is necessary given the genus’s size, these data will serve as a foundation for the potential elevation and diagnosis of the *Zanchius* group at the tribal level.

### ﻿Key to the *Zanchius* species in Vietnam

**Table:** 

1	Second antennal segment bicolorous or tricolorous with clear annulations (Fig. 1A–C, G, H)	**2**
–	Second antennal segment unicolorous (Fig. 1D, E, I, J)	**3**
2	Dorsum covered with two types of setae, silvery and dark setae; second antennal segment with dark and reddish annulations; corium with small marmorated greenish spots; diameter of a greenish spot much smaller than compound eye width, subequal to first antennal segment diameter; inner part of corium with dark spot; cuneus with silvery and dark setae; cells on membrane with dark spot (Fig. 1A–C); left paramere bifurcated; endosoma with three spiculi (Fig. 2A, E, F, M)	***Zanchiustuehang* sp. nov.**
–	Dorsum covered with silvery setae only; second antennal segment with dark coloration only; corium with large greenish markings; diameter of a greenish marking more or less compound eye in width; inner part of corium green; cuneus with silvery setae only; cells on membrane with green marking (Fig. 1G, H); left paramere scythe-shaped, not bifurcated; endosoma with two spiculi (Fig. 2B, G–I, N)	***Zanchiusmarmoratus***
3	Dorsum greenish with five noticable orange-red spots; first antennal segment clearly orange red; third antennal segment subequal to second antennal segment; apical part of clavus green; inner part of corium green; vein with a tiny dark spot (Fig. 1I, J)	***Zanchiusquinquemaculatus***
–	Dorsum mostly greenish with pale part; first antennal segment greenish pale brown; third antennal segment clearly shorter than second antennal segment; apical part of clavus pale brown; inner part of corium pale brown; vein unicolorous (Fig. 1D–F)	***Zanchius* sp.**

**Figure 1.**
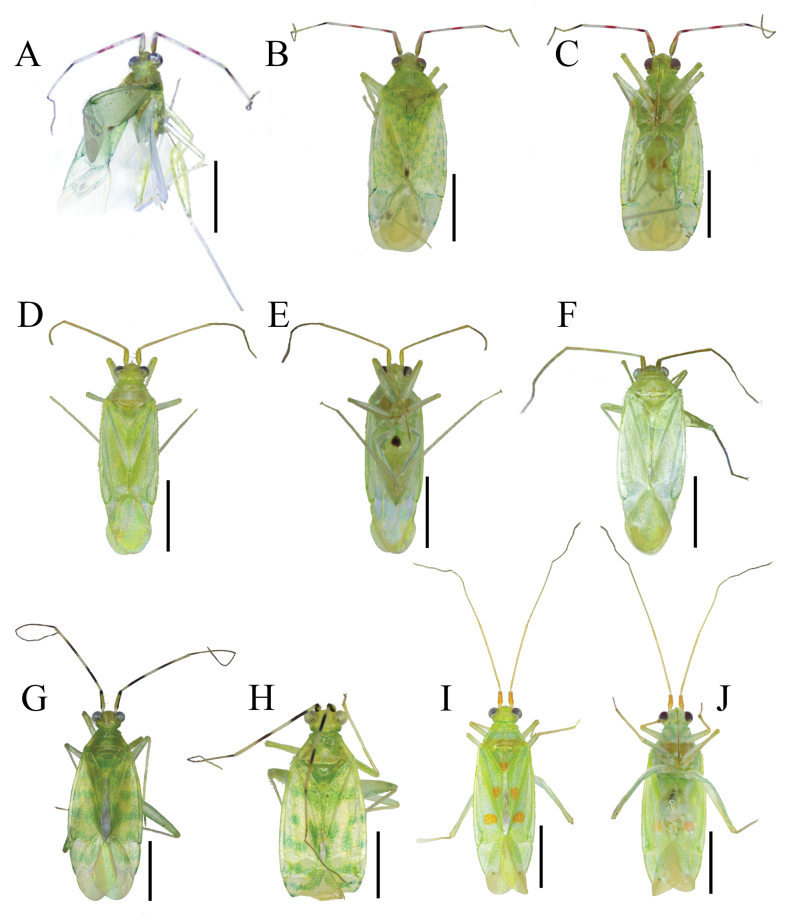
Habitus images of *Zanchius* spp. **A–C***Zanchiustuehang* sp. nov. **D–F***Zanchius* sp. **G, H***Zanchiusmarmoratus***I, J***Zanchiusquinquemaculatus***A** holotype, male **B, C** paratype, female in dorsal and ventral view **D, E** holotype, female in dorsal and ventral view **F** paratype, female **G** male **H** female **I** male in dorsal view **J***ditto*, in ventral view. Scale bars: 1 mm.

**Figure 2.**
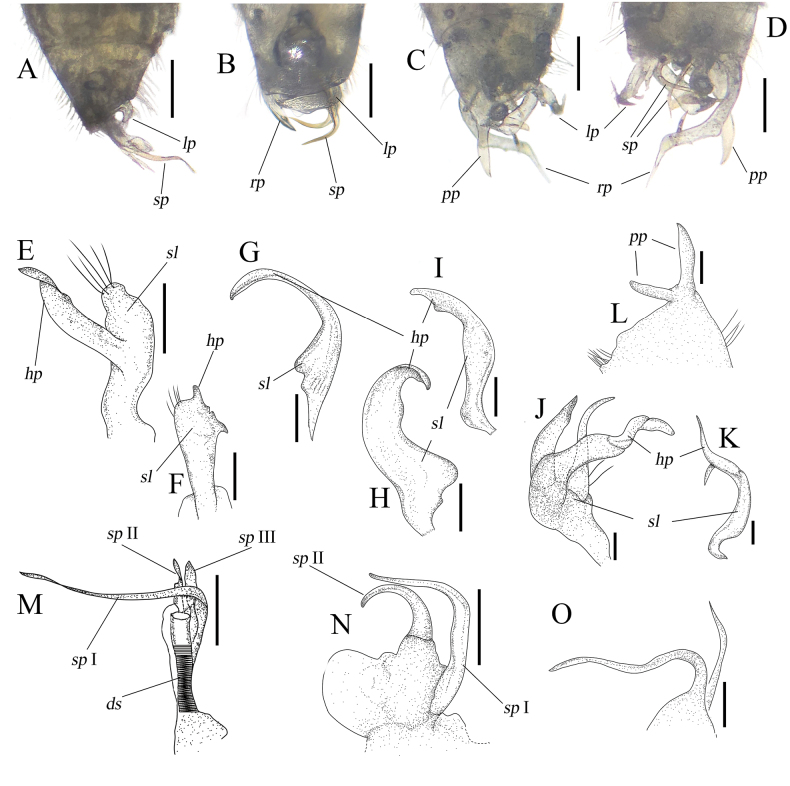
Pygopore and male genitalia of *Zanchius* spp. **A, E, F, M***Zanchiustuehang* sp. nov. **B, G, H, I, N***Z.marmoratus***C, D, J, K, L, O***Z.quinquemaculatus***A–D** pygopore **E, G, H, J** left paramere **F, I, K** right paramere **L** pygoporal process **M–O** endosoma. *hp*, hypophysis; *lp*, left paramere; *pp*, pygoporal process; *rp*, right paramere; *sl*, sensory lobe; *sp*, spicule. Scale bars: 0.2 mm (**A–D**); 0.1 mm (**E–O**).

**Figure 3.**
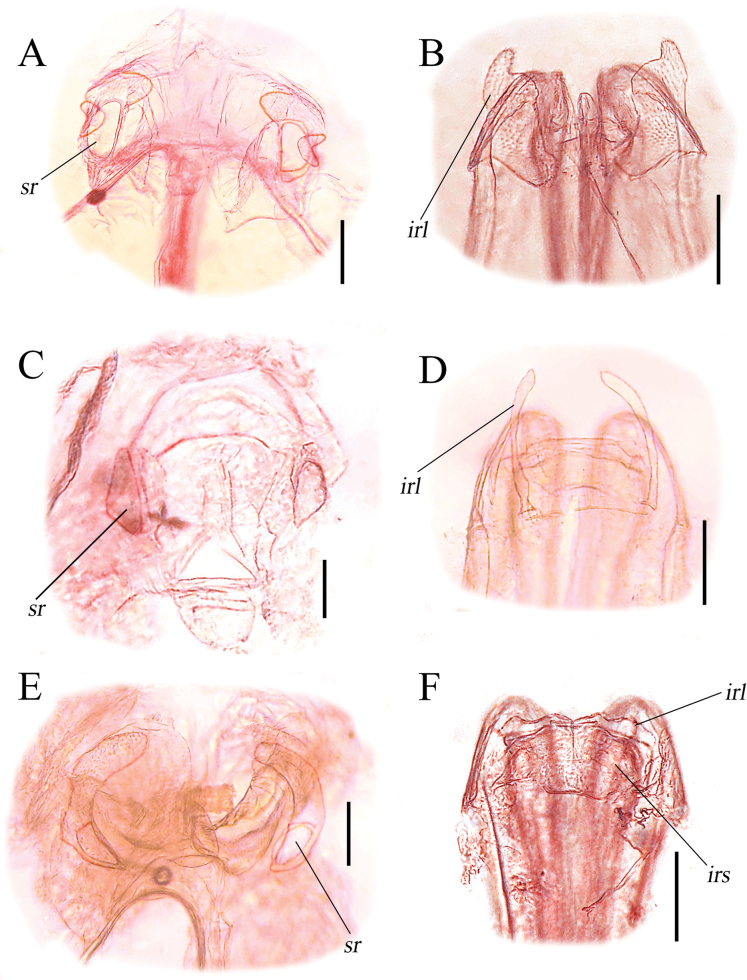
Female genitalia of *Zanchius* spp. **A, B***Z.tuehang* sp. nov. **C, D***Z.* sp. **E, F***Z.marmoratus***A, C, E** bursa copulatrix **B, D, F** posterior wall. *irl*, interramal lobe; *irs*, interramal sclerite; *sr*, sclerotized rings. Scale bars: 0.2 mm.

### ﻿ Zanchius tuehang

Taxon classificationAnimaliaHemipteraMiridae

Kim & Jung sp. nov.

C60135B8-3A7C-5078-8B5A-B2050C915311

https://zoobank.org/41F377CA-C588-4387-85EF-8D95879CD983

Figs 1A–C , 2A, E, F, M , 3A, B

#### Type materials.

***Holotype***: 1♂ Vietnam • Hoa Trung Lake, Hoa Lien Ward, Hoa Vang District, Da Nang City, 16.0891°N, 108.0425°E, 90 m altitude, on *Mallotusbarbatus* Müll.Arg., 1865, 2.xi.2024, J. Kim (ZCDTU) (DTUHMM0018). ***Paratypes***: Vietnam • 3♀♀, same data as for holotype (ZCDTU) (DTUHMM0019–0021).

#### Diagnosis.

Recognized by body mostly yellowish green with small, mottled, bluish-green spots, sparsely covered with long, dark setae; head mostly greenish pale brown; antennae with dark, reddish annulations, shorter than body length; first segment greenish pale brown, with lateral dark stripe, apically tinged with red, thickest; second segment pale brown with reddish annulations at base and on middle parts, with dark annulation at base, three times subequal to first segment; third and fourth segments pale brown with dark annulation basally; pronotum entirely green, with long dark setae sparsely at posterior part; scutellum yellowish pale brown, with long dark setae sparsely medially; hemelytra mostly yellowish brown with small mottled spots; clavus with long, dark setae, more sparse posteriorly; inner suture of corium with dark marking, with sparse, long, dark setae on dark marking; membrane subhyline, yellowish; cells with greyish marking at posterior part; legs mostly greenish; femur entirely green; tibia mostly pale brown, basal part greenish; left paramere bifurcated, hypophysis slightly coiled apically; right paramere simple, rod-shaped, with lateral spine; endosoma with three spiculi, one spicule long and two spiculi short, sharp (Fig. 2E, F, M).

#### Description.

**Male: *coloration*: head**: mostly pale brown with dark markings; vertex and frons yellowish pale brown; postocular part yellowish green; clypeus, juga and antennal socket greenish pale brown; antennae mostly pale brown with dark and reddish annulations; first segment pale brown with dark lateral stripe and reddish apical part; second segment pale brown with dark base and two medial reddish annulations, basal annulation close to dark base; third segment mostly pale brown with dark basal part; fourth segment mostly brown; labium entirely greenish pale brown. **Thorax**: pronotum mostly green, lateral areas and medial part somewhat paler; scutellum mostly green, somewhat pale green; hemelytra yellowish brown with tiny mottled greenish spots; clavus yellowish green with overall tiny greenish spots; corium yellowish brown with tiny greenish spots, anterior and posterior parts with relatively sparse spots, inner area with dark marking; cuneus subhyline, its margin bluish green; membrane greyish; vein tinged with green; two cells with dark spot posteriorly; legs mostly green; femur entirely green; tibia entirely pale brown, basal part somewhat green; tarsus brown, except for dark claw. **Abdomen**: entirely green. ***Surface and vestiture***: body shining, densely covered with two different types of setae, silvery and dark setae; head glossy; vertex and frons covered with silvery setae; first antennal segment covered with short erected dark setae; pronotum sparsely covered with short silvery setae laterally, and covered with dark setae posteriorly; scutellum sparsely covered with dark setae medially; hemelytra covered with two types of setae, silvery and dark setae; clavus covered with silvery setae, sparsely covered with dark setae along commissure; corium covered with silvery setae, middle part of corium sparsely covered with dark setae; embolial margin densely covered with short setae; cuneus sparsely covered with silvery setae, sparsely covered with dark setae along cuneal margins. ***Structure***: Body elongate-oval, length 2.91 mm. **Head**: hypognathous, two times as wide as long; vertex about as wide as eye; frons distinctly protruding forward of compound eye in dorsal view; postocular distance longer than first antennal segment diameter; antennae linear, shorter than body length; first segment thick, shorter than head length, longer than vertex width; second segment subequal to three times first segment length, subequal to combination of third and fourth segments; third segment distinctly longer than fourth segment; proportion of first to fourth antennal segments 0.3: 1.0: 0.5: 0.4; labium reaching hindcoxae, not reaching abdomen; proportion of first to fourth labial segments 0.3: 0.2: 0.17: 0.15. **Thorax**: pronotum trapezoid, basally twice wider than long, posterior margin concave; calli somewhat swollen; both scutellum and exposed part of mesoscutum large, combined length mesially shorter than wide, longer than pronotum; exposed part of mesoscutum large, slightly as long as scutellum at midline; commissure longer than scutellum; cuneus large, elongate, cuneal length laterally about one third as long as corial length; hindtibia shorter than costal margin. **Abdomen**: tapered to apex, not reaching apex of cuneus. **Genitalia**: pygophore asymmetrical with straight and angled margins (Fig. 2A); left paramere bifurcate, sensory lobe (*sl*) thick and blunt with long setae apically, hypophysis (*hp*) originating on middle part of sensory lobe, slightly coiled apically, apex thin (Fig. 2E); right paramere rod-shaped, *hp* short, simple, with one spine laterally (Fig. 2F); endosoma with three spiculi; one spicule (*sp* I) long and thin; two spiculi (*sp* II, III) short, subequal to 1/2 longest spicule, sharp, ductus seminis (*ds*) long (Fig. 2M).

**Female: *coloration***: as in male. ***Surface and vestiture***: as in male. ***Structure***: as in male, except for slightly wider vertex, exceeding compound eye width. **Genitalia**: sclerotized rings (*sr*) of dorsal labiate plate oval, with serrate areas (Fig. 3A); posterior wall with large, serrate interramal lobe (*irl*), apically terminating with blunt process (Fig. 3B).

#### Etymology.

Named after Dr Le Nguyen Tue Hang, Vice Director of Duy Tan University, in recognition of her invaluable support for this research; used as a noun in apposition.

#### Host.

*Mallotusbarbatus* (Euphorbiaceae).

#### Distribution.

Known only from the type locality.

#### Remarks.

This new species is most similar to the Japanese *Z.nakatanii* Yasunaga, 1999, but it can be distinguished by the following: dorsum being densely covered with tiny greenish spots (vs sparsely distributed in the latter species); the third antennal segment clearly longer than fourth segment (vs third segment subequal to fourth segment); clavus with dark setae (vs clavus without dark setae), small tiny greenish spots entirely distributed on clavus (vs small spots sparsely distributed on clavus); and cuneus with dark setae (vs cuneus without dark setae); see the original description by Yasunaga (1999) and fig. 52 by Yasunaga and Takai (2001). Yasunaga (1999: 171) presented “many green circular spots on the hemelytra” as a diagnostic character of *Z.nakatanii*, but the new species *Z.tuehang* sp. nov. exhibits denser spots than *Z.nakatanii*.

This new species also resembles the Taiwanese *Z.formosanus* Lin, 2005, but it can be distinguished by the following: small body, approximately 3 mm (vs larger body, about 3.3 mm); dorsum with tiny greenish spots (vs dorsum without tiny greenish spots); third antennal segment longer than fourth segment (vs third segment subequal to fourth segment); left paramere with long hypophysis and broad sensory lobe (vs left paramere with short hypophysis and narrow sensory lobe); and right paramere with short hypophysis and broad sensory lobe (vs right paramere with long hypophysis and not broad sensory lobe) (see Lin 2005: figs 1C, 2D–F).

### ﻿ Zanchius

Taxon classificationAnimaliaHemipteraMiridae

sp.

CB97A840-8780-52EC-8AE2-6CD036C3AF35

Figs 1D–F , 3C–D

#### Specimen examined.

1♀ Vietnam • Hoa Trung Lake, Hoa Lien Ward, Hoa Vang District, Da Nang City, 16.0891°N, 108.0425°E, 90 m altitude, on *Mallotusbarbatus*, 2.xi.2024, J. Kim (ZCDTU) (DTUHMM0022); 3♀♀ Vietnam • same location as for holotype, on *Mallotusbarbatus*, 7.xi.2024, J. Kim (ZCDTU) (DTUHMM0041–0043).

#### Description.

**Female: *coloration*: head**: entirely yellowish pale brown, except for the greenish posterior margin of head; antennae mostly light brown, slightly tinged with red-orange; first and second segments entirely light brown, slightly tinged with red orange; third and fourth segments brown, somewhat fuscous; labium greenish brown. **Thorax**: pronotum mostly green with medial pale marking; scutellum green, lateral areas and apex pale; hemelytra mostly green with pale parts; clavus mostly green, apical part pale; corium mostly green, posteroinner part pale, posterior part of embolium pale, with cell-like, green, oval margin; cuneus subhyaline, lateral margin green; membrane subhyaline, tinged with green basally; legs partly green and pale brown; femur mostly green; bluish-green stripe at lateral margin; tibia and tarsus entirely pale brown, except for dark claw. **Abdomen**: entirely green. ***Surface and vestiture***: body somewhat glossy, sparsely covered with long, silvery setae; head smooth; frons and clypeus densely covered with silvery setae; antennae densely covered with short, dark, erect setae; pronotum smooth, covered with setae in lateral margins; scutellum smooth; hemelytra covered with silvery setae; corium sparsely covered with silvery setae; lateral margin of embolium densely covered with silvery setae; cuneus covered with silvery setae. ***Structure***: body elongated, length 2.71–2.74 mm. **Head**: hypognathous, about twice as wide as long; vertex wider than eye; frons protruding forward of compound eye in dorsal view; postocular distance shorter than first antennal segment diameter; antennae linear, shorter than body length; first segment thick, shorter than head length, subequal to vertex width; second segment more than 5 times as long as first segment, subequal to third and fourth segments combined; third segment subequal to fourth segment; proportion of first to fourth antennal segments 0.2: 1.1: 0.6: 0.5; labium exceeding hind coxae; proportion of first to fourth labial segments 0.2: 0.19: 0.22: 0.34. **Thorax**: pronotum trapezoidal, basally twice wider than long, posterior margin sinuate; calli swollen; scutellum and exposed part of mesoscutum large, combined length mesially shorter than wide, longer than pronotum; exposed part of mesoscutum large, slightly as long as scutellum at midline; commissure about twice as long as scutellum; cuneus small, not elongate, cuneal length laterally about one-fourth as long as corial length; hind-leg remarkably long, hindtibia subequal to costal margin. **Abdomen**: tapered to apex, not reaching to apex of cuneus. **Genitalia**: sclerotized rings (*sr*) of dorsal labiate plate roughly triangular (Fig. 3C); posterior wall with serrate interramal lobe (*irl*), *irl* narrow, long, and apically rounded (Fig. 3D).

#### Host.

*Mallotusbarbatus* (Euphorbiaceae).

#### Distribution.

Known only from the specimen locality in Vietnam (Central).

#### Remarks.

Despite exhibiting distinct external morphology and female genital structures, we have decided to postpone its description as a new species until a male specimen becomes available, to avoid potential taxonomic issues because in most species studied in *Zanchius* so far, the male genitalia are well described, whereas information on the female genitalia is almost entirely lacking, with only a few species documented. Although the genus *Zanchius* may be subject to future revision, assigning this species to *Zanchius* is reasonable based on the currently established definition of this genus.

This species is most similar to the Taiwanese *Zanchiusapicalis* Poppius, 1915, but it can be distinguished by small body, approximately 2.7 mm (vs body large, approximately 4 mm); broader head, head length subequal to 1/2 head width (vs length more than 1/2 width); first antennal segment shorter than 1/5 second segment (vs first longer than 1/5 second segment); second antennal segment without apical marking (vs second segment with dark apical marking); and entirely green middle part of corium (vs middle part with pale marking) (see Lin 2005: figs 1A, B, 2A–C).

This species is similar in general shapes of body and head to *Zanchiusquinquemaculatus*, but it can be easily distinguished by body small (vs body large, more than 3 mm), without any colorful spots (vs with remarkable five orange-red spots); second antennal segment shorter than third segment (vs second subequal to third segment); inner part of corium with pale marking (vs inner part entirely green); and vein without any marking (vs vein apically with dark marking).

### ﻿ Zanchius marmoratus

Taxon classificationAnimaliaHemipteraMiridae

Zou, 1987

FBA06430-5AA8-5EA8-967A-6F3C857DE003

Figs 1G, H , 2B, G–I, N , 3E, F

Zanchius marmoratus Zou, 1987: 297, 299; Liu and Zheng 2014: 216.
Zanchius zoui Zheng & Liu, 1993:17 (syn. by Lin 2005: 189; Liu and Zheng 2014: 216).

#### Specimen examined.

Vietnam • 7♂♂10♀♀, Hoa Trung Lake, Hoa Lien Ward, Hoa Vang District, Da Nang City, 16.0891°N, 108.0425°E, 90 m altitude, on *Mallotusbarbatus*, 2.xi.2024, J. Kim (ZCDTU) (DTUHMM0023–0039).

#### Diagnosis.

Recognized by yellowish-brown body with bluish-green spots, covered with short, silky pubescence; head mostly pale brown, except for greenish posterior margin; antennae pale brown, with dark stripes and annulations, subequal to body length; first segment with dark stripe on outer lateral margin, longer than vertex width; second segment pale brown, with two dark annulations on subbasal and subapical parts; third segment pale brown with dark base; pronotum partly green and pale brown, lateral and posterior parts pale; scutellum mostly green, with pale apices; hemelytra subhyaline, yellowish pale brown, with bluish-green markings, covered with short silvery pubescence; clavus mostly green by having a large, greenish marking; corium mostly yellowish pale brown with three or four markings, inner and posterior margin greenish; cuneus mostly subhyaline, middle and apical part green; legs mostly yellowish brown; all femora yellowish brown with lateral bluish-green stripe; pygophore asymmetrical and posterior margin rounded; parameres scythe-shaped, endosoma with two sclerotized curved and long spiculi (Fig. 2G–I, N).

#### Description.

**Male**: see the original description by Zou (1987). **Genitalia**: pygophore asymmetrical with rounded posterior margin (Fig. 2B); left paramere scythe-shaped, sensory lobe broadened basally, hypophysis slightly broadened, rounded, its apex sharp (Fig. 2G, H); right paramere curved subapically, hypophysis with small projection medial inner part (Fig. 2I); endosoma with two sclerotized curved spiculi; spicule I (*sp* I) long and thin, vertically curved, gradually tapered from base; spicule II (*sp* II) shorter than *sp* I, extremely curved, rapidly tapered, its base remarkably broad (Fig. 2N).

**Female: *coloration***: as in male. ***Surface and vestiture***: as in male. ***Structure***: as in male, except for long antennae, more than body length. **Genitalia**: sclerotized rings (*sr*) of dorsal labiate plate narrow, with large and rounded serrate areas (Fig. 3E); posterior wall with simple interramal lobe (*irl*), *irl* contiguous, slightly expanded from interramal sclerite (*irs*), outer margin of *irl* sinuate (Fig. 3F).

#### Host.

*Mallotusbarbatus* (Euphorbiaceae)*.

#### Distribution.

Vietnam (Central)*, China (Southeast, Southwest), Taiwan.

#### Remarks.

Zou (1987) included drawings of the left paramere in two different orientations in the original description of this species. However, based on our direct examination of multiple specimens, we conclude that these drawings were horizontally flipped. Our description aligns with the illustrations provided by Lin (2005).

### ﻿ Zanchius quinquemaculatus

Taxon classificationAnimaliaHemipteraMiridae

Zou, 1987

0DD44605-4B3F-5449-A11B-41DA2C784B9E

Figs 1I, J , 2C, D, J–L, O

Zanchius quinquemaculatus Zou, 1987: 298, 300; Liu and Zheng 2014: 216.

#### Specimen examined.

Vietnam • 1♂, Hoa Trung Lake, Hoa Lien Ward, Hoa Vang District, Da Nang City, 16.0891°N, 108.0425°E, 90 m altitude, on *Mallotusbarbatus*, 2.xi.2024, J. Kim (ZCDTU) (DTUHMM0040).

#### Diagnosis.

Recognized by greenish body with five orange-red markings; head width wide; long antennae, more than body length; first segment mostly orange-red, short, subequal to vertex; second segment entirely reddish brown; third segment long, subequal to second segment; apical part of third segment and fourth segment dark brown; pronotum entirely greenish brown, calli region distinctly swollen; apex of scutellum with orange-red spot; hemelytra entirely green except for four orange-red spots; middle part of clavus with two red-orange spots; subposterior part of corium with two red-orange spots; cuneus entirely green; membrane entirely greyish; vein with dark small spot apically; femur entirely green; pygophore with two long noticable projections; parameres large; left paramere trifurcated; right paramere long with one sharp process subapically, its hypophysis sharp; endosoma with two long and sinuate spiculi (Fig. 2J–L, O).

#### Description.

**Male**: see the original description by Zou (1987). **Genitalia**: pygophore asymmetrical with two remarkable projections (Fig. 2C, L); left paramere trifurcated, sensory lobe with two long projections, hypophysis sinuated, its apex curved and blunted (Fig. 2J); right paramere long and curved, hypophysis sharp, with sharp process subapically (Fig. 2K); endosoma with two long and sinuate spiculi (Fig. 2O).

**Female**: not examined. According to Liu and Zheng (2014), one female specimen was examined without any comments, which may suggest no sexual dimorphism considering the unique and remarkable character of male.

#### Host.

*Mallotusbarbatus* (Euphorbiaceae)*.

#### Distribution.

Vietnam (Central, Northern), China (Southeast, Southwest).

#### Remarks.

In the present study, the relatively long third antennal segment, subequal to the second segment, is identified as a diagnostic character of this species, despite most members of *Zanchius* typically exhibiting the longest second antennal segment. This conclusion is drawn notwithstanding the antennal segment measurement for *Z.quinquemaculatus* (antennal segment lengths = 0.25:1.25:0.8:0.8) reported in the original description by Zou (1987), which differ from our observation. Furthermore, our direct examination revealed two spiculi in the endosoma, whereas only one spicule was described by Zou (1987). We infer that one spicule was overlooked in Zou’s (1987) description.

##### ﻿Notes on habitat sharing

**Figure 4.**
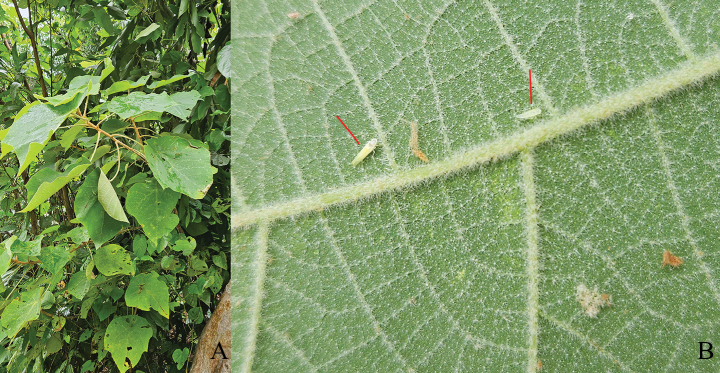
Host plant of *Zanchius* spp. **A***Mallotusbarbatus***B** its assumed prey (red arrows; probably typhlocybinid leafhopper species).

In our investigation, the four species of the genus *Zanchius* were observed to be exclusively associated with the host plant *Mallotusbarbatus* (Euphorbiaceae), which had been damaged solely by auchenorrhynchan species (Fig. 4). This suggests that the presence of *Zanchius* species on the host plant may be linked to the damage caused by the phytophagous species, which likely serve as prey for these opportunists. While other plant species exhibited signs of damage from auchenorrhynchans, *Zanchius* species were found only on specific host plants where both the damage and the phytophagous insects were present. This may indicate a strong host plant association, in which damage by auchenorrhynchans plays a role in attracting *Zanchius* species, possibly due to the availability of prey or specific ecological conditions provided by these plants (Messelink et al. 2012). Previous studies have reported that other *Zanchius* species (e.g. *Z.tarasovi* Kerzhner, 1988) are also found on host plants affected by auchenorrhynchan species, indicating that this association is not unique to the species observed in our study (Yasunaga 1999).

Among the four species of the genus *Zanchius* observed in this study, *Z.marmoratus* was collected in significantly greater numbers compared to the other species and is known to have a wider geographical distribution. These findings suggest that *Z.marmoratus* may possess a competitive advantage over the other species when sharing the same host plant. Moreover, given the extensive distribution of the host plant, *Mallotusbarbatus* (rainging from India to China), it is plausible that *Z.marmoratus* (or the genus *Zanchius* as a whole) has the potential for a broader geographic range.

While our observations confirm a strong association between the four *Zanchius* species and *Mallotusbarbatus*, the possibility of a broader host plant range should not be overlooked. Some *Zanchius* species, including *Z.tarasovi*, have been documented on phylogenetically diverse plant lineages, which may suggest a connection to the zoophytophagous nature of the genus. Given this broader host association in other *Zanchius* species, the four species examined in this study may also utilize additional host plants, despite our current observations being limited to *Mallotusbarbatus*. Further investigations are needed to determine the extent of their host plant flexibility and its ecological implications.

## Supplementary Material

XML Treatment for Zanchius

XML Treatment for Zanchius tuehang

XML Treatment for Zanchius

XML Treatment for Zanchius marmoratus

XML Treatment for Zanchius quinquemaculatus

## ﻿Additional information

### Author ORCIDs

Junggon Kim https://orcid.org/0000-0003-0594-7618

Tosaphol Saetung Keetapithchayakul https://orcid.org/0000-0001-7565-4701

Quoc Toan Phan https://orcid.org/0000-0002-3154-6546

Sunghoon Jung https://orcid.org/0000-0001-6086-0326

### Data availability

All of the data that support the findings of this study are available in the main text.
